# Complete Genome Sequence of a Copper-Resistant Bacterium from the Citrus Phyllosphere, *Stenotrophomonas* sp. Strain LM091, Obtained Using Long-Read Technology

**DOI:** 10.1128/genomeA.01327-16

**Published:** 2016-12-15

**Authors:** Damien Richard, Claudine Boyer, Pierre Lefeuvre, Olivier Pruvost

**Affiliations:** a Cirad, UMR PVBMT, St.-Pierre, Réunion, France; b ANSES, Plant Health Laboratory, St.-Pierre, Réunion, France; c Université de la Réunion, UMR PVBMT, St.-Denis, Réunion, France

## Abstract

The *Stenotrophomonas* genus shows great adaptive potential including resistance to multiple antimicrobials, opportunistic pathogenicity, and production of numerous secondary metabolites. Using long-read technology, we report the sequence of a plant-associated *Stenotrophomonas* strain originating from the citrus phyllosphere that displays a copper resistance phenotype.

## GENOME ANNOUNCEMENT

The genus *Stenotrophomonas* (*Gammaproteobacteria*) is composed of ubiquitous bacteria mostly found associated with plants and soil (1). Members of the genus *Stenotrophomonas* are known for their wide ecological niche range and their great phenotypic versatility including opportunistic pathogenicity to humans and resistance to many antimicrobials. For *S. maltophilia*, this high adaptability has been associated with a large number of genomic islands differing between strains and conferring them various adaptive traits (1).

Here, we sequenced the *Stenotrophomonas* sp. strain LM091 that was collected in Réunion (South West Indian Ocean) from the citrus phyllosphere. The strain possesses a copper-resistant phenotype genetically related to that reported for different plant pathogens in the *Xanthomonas* genus (2).

Sequencing was operated with PacBio RSII, using one single-molecule real-time (SMRT) cell. Resulting raw reads were assembled *de novo* using SMRT Analysis HGAP v2.3 protocol with default parameters followed by a circularization step based on a combination of the minimus assembler (3) and the SMRT Analysis resequencing v1 protocol. The assembly yielded a single circularized contig of 4,317,450 bp with a G+C content of 66.7%, a mean coverage of 229, and a predicted number of coding sequences (CDS) of 3,800.

The 16S rDNA sequence was 100% identical to that of *S. rhizophila* strain QL-P4 and 99.8% identical to that of *S. rhizophila* type strain DSM14405. As advised in Figueras et al. (4), we calculated the average nucleotide identity (ANI) (program available at https://github.com/DamienFr/) scores with all the chromosome sequences of type *Stenotrophomonas* strains available to date. As the ANI scores were always inferior to the 95% species ANI cutoff value (ranging from 80.2 to 87.6%), this strain could not be assigned to any of the valid *Stenotrophomonas* species nor to putative new species recently studied (5). The most closely related species were *S. rhizophila* type strain DSM14405 (87.6%) and *S. maltophilia* type strain MTCC434 (84.3%).

LM091 comprises a 43-kb long chromosome-borne Tn3-like transposon encoding for the *copLAB* gene system. This system, demonstrated to confer a copper-resistant phenotype, was previously identified on plasmids from several *Xanthomonas* (6). In Réunion, this adaptive transposon was also found in the same niche in the citrus pathogen *Xanthomonas citri* pv. *citri*, suggesting putative transfers of genetic elements between these two genera, as previously proposed (7). The nucleotide sequence of the copper-resistance transposon of LM091 is 97.7% identical to that of *Xanthomonas citri* pv. *citri*. The sequence presented here is part of the sequencing effort necessary to further understand the putative implication of strains of *Stenotrophomonas* in the transfer of genes conferring resistance to antimicrobials.

### Accession number(s).

The genome sequence has been deposited at GenBank under the GenBank accession no. CP017483.
